# The use of biologics to improve patient-reported outcomes in hip preservation

**DOI:** 10.1093/jhps/hnab028

**Published:** 2021-04-17

**Authors:** Spencer W Sullivan, Oluwatobi M Aladesuru, Anil S Ranawat, Benedict U Nwachukwu

**Affiliations:** 1 Sports Medicine Institute, Hospital for Special Surgery, 610 West 58th Street, 3rd Floor, New York, NY 10019, USA; 2 Weill Cornell Medical College, New York, NY, USA

## Abstract

Despite lack of clear understanding, the use of biologic treatment methods has increased in the United States. Therapeutic methods, including platelet-rich plasma (PRP), bone marrow aspirate concentrate (BMAC) and hyaluronic acid (HA) among other biologics, are commonly associated with relief of pain in a number of different orthopedic conditions. Within the past two decades, hip preservationists have investigated the roles of these biologic treatments in both non-operative and surgical management of common hip conditions. The purpose is to review the published literature surrounding the application and efficacy of biologics, most notably PRP, BMAC and HA, in the clinical management of hip conditions. The hip conditions examined in this review include hip osteoarthritis, femoroacetabular impingement syndrome and associated labral tear pathology, avascular necrosis of the femoral head and gluteal/hamstring tendinopathy. While our review of the literature suggests that there is support for the implementation of biologics to relieve pain and improve function for hip conditions. Through further research efforts, it is important to stay updated with the clinical efficacy of biologics in hip preservation.

## INTRODUCTION

Despite lack of clear understanding of their clinical efficacy and cost-effectiveness, the clinical use of biologic therapies has increased in the United States in recent years [1–3]. Animal studies have verified ‘proof of concept’ for cell-based therapies, platelet-rich plasma (PRP), cytokines and tissue-engineered implants but due to insufficient number of quality studies and lack of standardization in biologic preparations and outcomes, the clinical use for these therapies remains unclear [1, 3]. Direct-to-consumer advertising of biologic therapies by clinics and commercial entities portrays an almost exclusively positive tone, without providing a balanced view of the risks, benefits and limitations of these treatments. Because of this, physicians must be aware of the discrepancy between marketing claims and scientific evidence [1]. Concerns about such misinformation from direct-to-consumer marketing about unproven treatments have led several medical professional societies to convene and discuss the matter, as misrepresentation and lack of characterization of these products may erode public trust and interfere with the development of legitimate cell therapies [2]. Additionally, Turner and Knoepfler [4] identified 351 US centers that offer direct-to-consumer stem cell therapies at 570 clinics. These clinics were associated with an average cost of ∼$5000 for a biologic injection for knee osteoarthritis (OA) and purported an 80% patient satisfaction and symptomatic improvement rate, a statistic that differs from what is in the published literature [5].

The purpose of this article is to (i) review the published literature surrounding the application and efficacy of biologics, most notably PRP, bone marrow aspirate concentrate (BMAC) and hyaluronic acid (HA), in the clinical management of hip conditions and (ii) analyze the effects of biologic therapies on patient-reported outcomes in the literature. Articles were included in our literature review if they analyzed patient-reported outcomes and identified biologic therapies as a targeted treatment method for the following hip conditions: hip OA, femoroacetabular impingement syndrome (FAIS) and associated labral tear pathology, avascular necrosis of the femoral head, gluteus tears/tendinosis and hamstring tendinopathy (Table I).

**Table I:** Breakdown of research trials analysed in this review of biologics used in common conditions of the hip

Study	Study design	Patients (*N*)	Intervention	Condition	Patient group	Control group	Dose	Outcome measures	Follow-up	Findings
Qvistgaard *et al*.	RCT	101	HA	Osteoarthritis	Group 1: HA intra-articular injection	Saline injection	3 injections, 2 weeks apart	VAS for pain	2-week, 1-month, 3-month	There was no difference between groups at each endpoint but there was a small clinical improvement in the HA group
					Group 2: Corticosteroid injection					
Migliore *et al*.	RCT	42	HA	Osteoarthritis	HA intra-articular injection	Local anesthetic	2 injections, 1 month apart	Lequesne index, VAS for pain, Global Score	3-month, 6-month	Clinically significant improvements were found in the HA group within the Lequesne index and Pain scores at 3 and 6-month follow-up
Battaglia *et al*.	Pilot cohort study	20	PRP	Osteoarthritis	Ultrasound-guided PRP injections	—	3 injections, 2 weeks apart	HHS, WOMAC	1, 3, 6, 12-month	Significant improvements in HHS and WOMAC at each follow-up period were reported compared to baseline, however, these results were not sustained
Sanchez *et al*.	Prospective case series	40	PRP	Osteoarthritis	Ultrasound-guided PRP injections	—	3 injections, 1-2 weeks apart	HHS, WOMAC, VAS for pain	6-week, 6-month	Significant improvements in function and pain at each follow-up period were found compared to baseline; however, the PRP treatment was not effective in 27.5% of patients
Battaglia *et al*.	RCT	100	PRP, HA	Osteoarthritis	PRP intra-articular injection	HA intra-articular injection	1 injection	HHS, VAS for pain	1, 3, 6, 12-month	PRP injections are efficacious, but not significantly superior to HA injections at 12-month follow-up for improving pain and function
Dallari *et al*.	RCT	111	PRP, HA, combination of both	Osteoarthritis	Group 1: PRP intra-articular injection, Group 2: HA and PRP intra-articular injection	HA intra-articular injection	3 injections, 1 week apart	VAS for pain, WOMAC	2, 6, 12-month	PRP injections offer significant clinical improvement and slightly more stable at 12-month follow-up compared to other treatment methods tested. The combination of HA and PRP did not significantly reduce pain
Doria *et al*.	RCT	80	PRP, HA	Osteoarthritis	PRP intra-articular injection	HA intra-articular injection	3 injections, 1 week apart	HHS, WOMAC, VAS for pain	6-month, 12-month	PRP and HA injections were not significantly different in patient-reported measures of pain and function at each follow-up period
Rodriguez-Fontan *et al*.	Prospective case series	19	BMAC	Osteoarthritis	BMAC intra-articular injection	—	1 injection	WOMAC	6, 12, 18, 24-month	63.2% of patients reported satisfactory results following BMAC injections with significant improvement noted in WOMAC scores at follow-up
Abate *et al*.	Prospective case series	20	HA	FAI	HA intra-articular injection	—	3 injections over 6 months	VAS for Pain, HHS, Lequesne Index	6-month, 12-month	HA is effective in mild cases of FAI with significantly improved outcomes in disability, pain and function at 6 and 12 month follow-up
Rafols *et al*.	RCT	57	PRP	FAI	PRP intra-articular injection during hip arthroscopy	Hip arthroscopy without PRP	1 injection intra-operatively	VAS for Pain, mHHS	48-hour; 3, 6, 12, 24-month	PRP was found to benefit postoperative inflammation, but long-term benefits remain unclear in improvements of pain and function due to PRP
Lafrance *et al*.	RCT	35	PRP	FAI	PRP intra-articular injection during hip arthroscopy	Saline injection during hip arthroscopy	1 injection intra-operatively	mHHS, HOS, NAHS	1, 3, 6, 12-month	Intra-operative PRP injections did not significantly improve clinical outcomes compared to saline at 1-year follow-up from hip arthroscopy.
Rivera *et al*.	Prospective cohort study	40	BMAC	FAI	BMAC intra-articular injection during hip arthroscopy	—	1 injection intra-operatively	VAS for pain, mHHS, iHOT	12-month, 24-month	Patients reported significantly improved outcomes following hip arthroscopy with BMAC intra-operative injection at 2-year follow-up.
Redmond *et al*.	Prospective cohort study	271	PRP	FAI	PRP intra-articular injection during hip arthroscopy	Bupivacaine injection during hip arthroscopy	1 injection intra-operatively	VAS for pain, mHHS, HOS, NAHS	3-month, 2-year	For patients undergoing hip arthroscopy for labral repair, intraoperative PRP injections do not improve pain and functional outcomes
Mishima *et al*.	Prospective case series	14	BMAC	ONFH	BMAC grafting with low-intensity pulsed ultrasound	—	1 injection	VAS for Pain, JOA hip score	6, 12, 24-month	New bone formation was identified in all patients with increased pain and function outcome scores in all patients at final follow-up.
Pilge *et al*.	Matched pair analysis	20	BMAC	ONFH	BMAC injection during core decompression	Core decompression without BMAC injection	1 injection intra-operatively	Merle d'Aubigne Score	Inconsistent	There was no difference between functional outcomes between treatment groups. Patients given BMAC are less likely to undergo further procedures, including THA.
Pepke *et al*.	RCT	25	BMAC	ONFH	BMAC injection during core decompression	Core decompression without BMAC injection	1 injection intra-operatively	VAS for Pain, HHS	3, 12, 24-month	The addition of BMAC did not provide significant clinical benefits in patients undergoing core decompression clinically and radiographically
Cruz-Pardos *et al*.	Retrospective cohort study	60	BMAC	ONFH	BMAC injection during core decompression	Core decompression without BMAC injection	1 injection intra-operatively	Merle d'Aubigne Score	6, 12, 26, 52-week, then annually	This study could not determine outcome differences specifically due to the intra-articular injection of BMAC as the conclusion of core decompression
Hauzer *et al*.	RCT	38	BMAC	ONFH	BMAC injection during core decompression	Saline injection during core decompression	1 injection intra-operatively	VAS for Pain, WOMAC	3, 6, 12, 24-month	No significant differences were found in both functional and pain improvement as well as readiologic evidence between treatement methods
Fitzpatrick *et al*.	RCT	80	PRP	Gluteus Pathology	PRP injection into the affected gluteal tendons	Corticosteroid injection	1 injection	mHHS	2, 6, 12-week	Patients with PRP injections experienced significant improvements in pain and function at 12-week follow-up compared to the control group
Fitzpatrick *et al*.	RCT with cross-over	80	PRP	Gluteus Pathology	PRP injection into the affected gluteal tendons	Corticosteroid injection	1 injection	mHHS	12, 24, 104-week	Patients treated with a PRP injection continue to show greater functional and pain improvements than the steroid group >15-month follow-up
Lee *et al*.	Prospective cohort study	21	PRP	Gluteus pathology	PRP injection with needle tenotomy	—	1 injection	mHHS, HOS, iHOT	mean 19.7-month	PRP injections with needle tenotomy is safe and effective means for improving pain and function in patients experiencing gluteal pathology
Jacobson *et al*.	Prospective cohort study	30	PRP	Gluteus Pathology	PRP injection	Needle fenestration	1 injection	VAS for Pain	mean 8-day and 18-day	No significant difference in short-term pain scores were examined between treatment groups as both methods reduced pain scores
A Hamid *et al*.	RCT	24	PRP	Hamstring Pathology	PRP injection with rehabilitation program	Rehabilitation program only	1 injection	Subjective return to play	Inconsistent	Patients that received a PRP injection with their rehabilitation program were able to return to play significantly quicker than patients with only a rehabilitation program
Park *et al*.	Retrospective cohort study	56	PRP	Hamstring Pathology	PRP injection into the affected hamstring tendons	Steroid injection	1 injection	VAS for Pain	1-week and 4-week	Patients that received a PRP injection experienced better short-term pain relief compared to patients that received a steroid injection
Levy *et al*.	Prospective cohort study	29	PRP	Hamstring Pathology	PRP injection under ultrasound guidance	—	1 injection	VISA-H questionnaire	8-week	At 8-week follow-up, patients did not experience significant clinical outcome improvement in this pilot study

RCT, randomized controlled trial; HA, hyaluronic acid; PRP, platelet-rich plasma; BMAC, bone marrow aspirate concentrate; VAS, visual analog score; HHS, Harris hip score; WOMAC, Western Ontario and McMaster Universities Osteoarthritis Index; FAI, femoroacetabular impingement; ONFH, osteonecrosis of the femoral head; mHHS, modified Harris hip score; HOS, hip outcome score; NAHS, non-arthritic hip score; iHOT, international hip outcome tool; JOA, Japanese Orthopaedic Association; VISA-H, Victorian institute of sport assessment proximal hamstring tendons.

## BIOLOGIC OPTIONS IN ORTHOPEDIC SURGERY

### Platelet-rich plasma

PRP is a volume of autologous plasma that exhibits a platelet concentration above baseline levels and is rich in growth factors. Two methods have been described to prepare PRP: centrifugation and apheresis [6, 7]. Centrifugation produces PRP in a less expensive and more practical manner; however, apheresis yields a higher platelet concentration [6]. The rationale for use and therapeutic potential of a high concentration of platelets is based on their capacity to supply and release supraphysiologic amounts of essential growth factors and cytokines from their alpha granules to provide a regenerative stimulus that augments healing and promotes repair in tissues with low healing potential [7]. Some studies have shown that growth factors present in PRP were shown to increase tenocyte proliferation and extracellular matrix production while protecting against oxidative stress and inflammation [1].

In 2011, Nguyen *et al*. [8] performed a comprehensive review of the literature surrounding the therapeutic applications of PRP in musculoskeletal and sports medicine at the time. Many of the studies showed the potential positive effect of PRP in the treatment of musculoskeletal conditions, but, at the time, there was a paucity of human randomized controlled trials (RCTs) to provide evidence for its efficacy. Since then, several RCTs have been published supporting the effectiveness of PRP in specific musculoskeletal injuries, including rotator cuff tears and general tendinopathy [9–12]. Additionally, prior literature supports the intraoperative use of PRP injections during rotator cuff repair and anterior cruciate ligament reconstruction [1, 10, 11, 13]. However, other studies investigating the efficacy of PRP in other shoulder pathologies, including rotator cuff tear and tendinopathy, have countered the previous evidence of the benefits of biologics compared to standard of care treatments [1, 14, 15].

The lack of clarity surrounding PRP likely stems from the lack of standardization in its preparation and lack of characterization of the final product [1, 6]. Variation in characteristics, such as platelet concentrations, platelet activation, leukocyte concentrations and other contents, is dependent on numerous factors, including harvest site, harvest volume, patient demographics, such as age and sex, concomitant medical comorbidities, medications, nutritional factors and recent activity level [1]. PRP is an autologous product, therefore, biological difference among individuals and hematocrit variability can contribute to the observed variation in PRP content and quality, as well as, the cellular responses to autologous blood products and tissue-specific metabolic needs [1, 3, 6].

### Bone marrow aspirate concentrate

Stem cells can be extracted from the blood, bone marrow, fat, muscle and virtually every tissue in the body [16, 17]. BMAC contains growth factors that are linked to chondrocyte proliferation, mesenchymal stem cell (MSC) differentiation, wound healing and the suppression of proinflammatory cytokines [17]. Similar to PRP, the clinical efficacy of BMAC has also been debated in the orthopedic literature, and the optimal preparation, anatomic source, delivery method, timing and dosage remains largely unclear [18, 19]. Specifically, the viability and efficacy of BMAC and other cell therapies are affected by harvest site, cell concentration and donor characteristics, including age, sex and comorbidities [1]. Due to this lack of clarity and understanding around BMAC, it is important to conduct further investigation to help build a systematic algorithm based on characteristics of the patient and the pathology [1, 17].

The use of BMAC has been associated with osteochondral repair and a finding of significantly increased defect filling, better structural parameters of the repaired tissue and improved integration compared with microfracture alone in the ankle and the knee [20, 21]. Additionally, patients with knee OA who were given a single intra-articular BMAC injection appeared to have long-term benefits, with statistically significant improvement in pain and function [22]. Trials have also shown that outcomes using bone marrow-derived MSCs have been more positive than using PRP in the setting of rotator cuff repair [1]. Although there is evidence on the use of BMAC in symptomatic knee OA, no consensus exists on the algorithm for treatment, indications, optimal protocol of processing and delivery and outcome reporting in the current literature.

### Hyaluronic acid and other biologic therapies

An additional biologic therapy is the use of HA, a glycosaminoglycan primarily located in the cartilage extracellular matrix within a joint’s synovial fluid. HA primarily functions as a shock absorber within joints, such as the hip, in addition to its lubricating properties to allow for smooth motion of the femoral and acetabular surfaces [23]. Within the orthopedic literature, intra-articular injections of HA have been used as an additional lubricant for compromised joints to provide pain relief and improved joint function. Multiple studies have analyzed the effects of HA and found similar benefits compared to other non-operative management and operative techniques for pain relief of the hip and knee [24].

Additionally, a wide array of biologics are incorporated into orthopedic surgery and hip preservation surgery specifically, including chondrocyte implantation, MSCs, growth factors and cytokines as well as various biomolecular and tissue-engineered implants [25–27]. Krueger *et al*. [26] analyzed the effects of injecting autologous chondrocyte implantation into acetabular lesions and found promising results at 3-year follow-up. MSCs have additionally been shown to be safe and effective in the treatment of hip OA for relieving pain and restoring function and range of motion [27]. Additionally, other biologic therapies are emerging withing the United States for sports medicine and other orthopedic conditions [25]. However, due to the nature of this review and the paucity of research surrounding these additional biologics therapeutic methods, this review focuses on the implementation of PRP, BMAC and HA within hip preservation surgery.

### Hip osteoarthritis

In 2006, Qvistgaard *et al*. [28] performed an RCT comparing intra-articular injections of HA, corticosteroid, or isotonic saline in the treatment of hip OA. Patients treated with corticosteroids experienced significant improvement in pain while patients treated with HA did not have significant improvements. Despite three doses over the span of 6 weeks, the study found a small effect size between the treatment groups, thus warranting further research for the clinical efficacy of HA [28]. A subsequent RCT comparing the use of intra-articular HA with the intra-articular analgesic for treatment of hip OA found that intra-articular HA is safe and has beneficial effects in the management of hip OA [29]. Despite clinical effectiveness, this study had lower power with only 42 patients split between the two treatment arms. Additionally, Bowman *et al*. [30] performed a review of prospective clinical trials investigating the efficacy of HA injections in knee, hip and ankle OA. In their search, they found support for superior pain relief of HA injection over placebo in a number of studies. They further concluded that several trials found HA to be more effective than PRP in treatment of patients with mild to moderate hip OA, but there is no clear evidence to prove its ability to modify the morphological or radiological changes of the pathological hip [30]. While it seems HA has been shown to provide relief and is considered safe and efficacious to use for hip OA, there may be other treatment methods that provide longer-lasting relief. These studies have shown that HA can provide relief in pain and function, but for HA injections specifically, outcomes up to 6-month follow-up have been analyzed with little support to determine endpoint treatment methods for osteoarthritic pain.

Preliminary studies evaluating the safety and efficacy of intra-articular PRP injections for hip OA found that administration of PRP is associated with clinical improvement in pain, regardless of the patient’s sex, age, body mass index or OA grade despite no comparison to a control group and a limited number of patients in both studies [31, 32]. Comparing the clinical efficacy of PRP versus HA, Battaglia *et al*. [33] later found that ultrasound-guided, intra-articular injections of PRP are as safe and efficacious as HA in improving function and reducing pain in a cohort of 100 patients at 12-month follow-up. A meta-analysis of four RCTs comparing the benefits of PRP versus HA in patients with hip OA found that while both PRP and HA were comparable in terms of functional recovery, PRP offered significant reductions in pain [34]. Dallari *et al*. [35] performed an RCT to compare the therapeutic efficacy of ultrasound-guided injections of autologous PRP, HA or a combination of both (PRP + HA) in hip OA. Their results indicated that intra-articular PRP injections offer significant clinical improvements in pain, function and quality of life in patients with hip OA, a benefit that was significantly more stable up to 12 months as compared with the other tested treatments. However, additional treatment of PRP + HA did not lead to a significant improvement in pain symptoms [35]. Another study by Doria *et al*. [36] performed an RCT comparing the clinical efficacy of ultrasound-guided intra-articular injections of PRP versus HA for treatment of early hip OA. Both groups showed significant improvements in pain and functional outcome measures at both 6- and 12-month follow-up, without any major complications. The authors concluded that, because PRP did not offer better results compared with HA, it should not be considered as the first-line treatment for hip OA [36].

With somewhat mixed results between these three RCT studies, each of these studies had a maximum of 111 patients enrolled in the study, regardless of treatment arm. Further investigations with a larger cohort could be beneficial to supporting PRP as an effective biologic for the general hip OA population. Additionally, standardization of the dosage of PRP is needed to help determine the long-term effectiveness of PRP. While three injections were utilized in both RCTs by Dallari *et al*. and Doria *et al*., two different conclusions were made at the 12-month follow-up regarding the efficacy of PRP compared to HA. Further evidence is needed to ultimately determine PRP’s use in the treatment of hip OA.

Compared to PRP injections, there is a paucity of research on the clinical efficacy of BMAC in the treatment of hip OA. Rodriguez-Fontan *et al*. performed a prospective cohort study, investigating the clinical outcomes of patients undergoing intra-articular injection of BMAC for treatment of early hip and knee OA. The outcomes in the study were unpredictable with a 63.2% patient-reported satisfaction rate [37]. However, this study was poorly designed without any control group and backed by low power with only 19 patients. This certainly warrants further research to support the efficacy of BMAC injections in the treatment of hip OA. Overall, based on the available literature—biologic options appear to be beneficial in the short term for the treatment of mild to moderate hip OA. Medium to long-term data (>12-month outcomes) is lacking in all forms of biologic therapy.

### Femoroacetabular impingement and labral tear pathology

Femoroacetabular impingement (FAI) was first described in the English literature in 1999 and is now considered one of the main causes of hip pain in the athletic population. More recently, FAI had been identified as a predictor of premature hip OA in the younger population [6]. FAI is defined as a painful conflict between the acetabular rim and the proximal femur during end-range hip motion [6]. While hip arthroscopy has become a primary treatment method for recurrent symptomatic pain due to FAI, intra-articular ultrasound-guided injection of HA has been suggested to be a safe and effective non-surgical treatment method. Abate *et al*. [38] found that intra-articular ultrasound-guided injection of HA significantly decreased pain and improved function in patients with mild FAI who are not yet candidates for surgery. Despite clinical effectiveness, only 20 patients received three injections over the span of six months. Higher powered studies and longer follow-up should be conducted in future research to support the efficacy found in HA in this specific study.

The application of biologics during arthroscopic FAI surgery has also been studied. An RCT examined the effects of intra-articular injection of PRP during hip arthroscopy with indication of FAI. The cohort treated with PRP intraoperatively (*N* = 30) reported lower pain scores 48 h post-operatively and demonstrated fewer joint effusions at 6-month follow-up. The study supported the use of intraoperative PRP intra-articular injections to reduce inflammation following hip arthroscopy but concluded more research should be conducted to determine long-term benefits [39]. Similarly, another RCT demonstrated decreased inflammation and ecchymosis following hip arthroscopy with intra-articular PRP injections. Despite immediate post-operative benefits, the study concluded that PRP injections did not produce significant improvements in outcomes at 1-year follow-up when comparing 20 patients who received PRP to 15 control patients without PRP intra-articular injections [40]. In addition to PRP, BMAC has been used intra-operatively for hip arthroscopy procedures with indication of FAI. A 2020 study supported that BMAC injections at the conclusion of the arthroscopy procedure improve outcomes of both pain and function at 1- and 2-year follow-up [41]. Interestingly, there was not a control group utilized in this study to determine how BMAC specifically improved outcomes. Without an arm of patients that underwent hip arthroscopy without BMAC intra-articular injections, it is difficult to support the isolated effect of BMAC on improvements in the visual analog score, modified Harris hip score and international hip outcome tool.

Within the context of hip labral repair, a 2015 prospective cohort study compared intraoperative PRP injections (*N* = 91) and bupivacaine injections (*N* = 180), and at 2-year follow-up, the study found no difference in both pain and functional outcome measures. Despite no statistical difference, the study group exhibited lower Harris hip score scores than the control group (78.6 versus 82.6, respectively). The study concluded that intraoperative PRP injections do not appear to provide clinical improvements in patients undergoing hip arthroscopy for labral pathology [42]. Interestingly, this study was well-powered with 306 patients included in the study compared to previous lower-powered studies specific to biologic use in FAI treatment. Additionally, a 2019 systematic review of biological therapies as an adjunct to hip arthroscopy for FAI similarly found that PRP was ineffective for labral tear pathology at short-term follow-up. The study further concluded that the quality of the literature is low regarding biological treatments for FAI and associated labral pathology and emphasized the lack of homogeneity in biologics research to accurately measure the clinical efficacy of these treatment methods [43]. A majority of the biologic literature in hip arthroscopy focuses on intra-articular injections of PRP and BMAC without much support for its efficacy in further improving long-term and short-term outcomes in most studies identified. However, there is support for reduced inflammation and pain in the immediate post-operative period when using a single intra-articular injection during a hip arthroscopy procedure. This may be important to mention in determining the optimal means of patient treatment in hip preservation for FAI.

### Avascular necrosis of the femoral head

Biological treatments have also been used to treat avascular necrosis of the femoral head. In a 2016 study, Mishima *et al*. described a novel therapy for osteonecrosis of the femoral head (ONFH) based on autologous bone marrow grafting and low-intensity pulsed ultrasound. In a cohort of 14 patients, the study concluded that there were improvements in pain and function with new bone formation, no infection of the grafted bone marrow and no tumor development at the treatment site. Of the 22 hips treated, 8 had progression of collapse, but none required total hip arthroplasty [44]. Additionally, Pilge *et al*. investigated the benefits of BMAC injection in combination with core decompression (CD) for ONFH using a matched pair analysis. In this study, patients who received BMAC in addition to CD had decreased pain, fewer joint symptoms, improved range of motion and slower progression of disease. The study also found that additional BMAC application prevents the collapse of the femoral head and significantly reduces the necessity of further surgery, including total hip replacement [45].

However, several studies have refuted clinically significant effects after biological treatment for ONFH. A randomized prospective cohort study followed 25 patients that underwent CD for atraumatic ONFH with 11 of the patients receiving an additional BMAC injection during the CD procedure. The BMAC-treated cohort demonstrated no benefit in clinical or radiological outcomes at 2-year follow-up [46]. Similarly, Cruz-Pardos *et al*. [47] studied the effectiveness of CD combined with injection of autologous bone marrow concentration for treatment of ONFH as compared to CD alone. The findings of the study confirmed the safety of CD and autologous bone marrow graft, but no significant difference in progression to femoral head collapse was noted. Finally, Hauzer *et al*. [48] also performed an RCT evaluating the effect of BMAC in addition to CD in stage 3 ONFH as compared to CD plus saline injection. Their findings showed no benefit over a control saline injection for pain and functional improvement, radiologic evolution and the eventual requirement of total hip replacement.

Although previous studies exemplified mixed results in the efficacy of a BMAC intra-articular injection alongside CD for avascular necrosis, hip preservationists often utilize BMAC in this patient population for various CD techniques. One important point to make is that each study examined retained relatively low power, often analyzing cohorts of around 20 patients. Additionally, variance in the efficacy of BMAC could have been attributed to multiple factors including stage of collapse of the femoral head. While BMAC may be useful for pre-collapsed patients, high-grade osteonecrotic lesions may be considered too advanced for BMAC, let alone CD, to be an effective treatment method before resorting to arthroplasty or resurfacing. Finally, it is important to standardize the outcomes that are assessed in this population. Standardization of outcome assessment and increasing the power of future studies will help to fortify the role of biologics, specifically BMAC, in treatment of ONFH.

### Gluteal tendinopathy and injury

Lateral hip pain, historically labeled as trochanteric bursitis, is now characterized under the category of greater trochanteric pain syndrome (GTPS) with many common pathologies [6]. Under the umbrella term of GTPS, gluteus medius/minimus tendinopathy is a common cause of lateral hip pain [49]. Despite the paucity of literature, biological treatment of gluteal tendinopathy has been supported in the literature. Fitzpatrick *et al*. conducted an RCT of 80 patients matched 1:1 to compare a single PRP injection with a single corticosteroid injection in the treatment of chronic gluteal tendinopathy. The study found that patients treated with a single PRP injection achieved greater pain and functional improvement at 12 weeks than those treated with a single corticosteroid injection. Additionally, a majority in the PRP group (82% compared to 56.7 and in the control group) achieved the minimal clinically important difference, a statistically significant difference [50]. A follow-up study published a year later investigated the effect of leukocyte-rich PRP injection for the treatment of chronic gluteal tendinopathy. Patients treated with a single leukocyte-rich PRP injection had greater improvements in pain and function and sustained improvement at 2-year follow-up, whereas improvement in pain and function was not sustained beyond 24 weeks in patients treated with a single corticosteroid injection [51]. Despite clinically significant improvements in the PRP injection group, there was crossover at 3 months post-operatively that allowed patients in the control group to receive a PRP injection. Although this study supported superior outcomes for PRP injections, it is important for further research to identify a similar pattern without patient crossover to determine the efficacy of this biologic therapeutic method.

PRP injections have also been paired with needle tenotomy and fenestration with patient reported, therapeutic success. A 2016 study treated 21 patients with chronic recalcitrant gluteus medius tendinopathy with ultrasound-guided intratendinous PRP injections and needle tenotomy. At a minimum 12-month follow-up, all outcome measures were clinically and statistically significant, and the researchers concluded PRP injections to be a safe and effective treatment method for gluteal tendinopathy [52]. However, with low patient recruitment and the absence of a control group, it is difficult to attribute outcome improvements to the PRP injection to the needle tenotomy which has achieved clinically significant outcomes alone in the hip preservation literature. To combat this limitation, Jacobson *et al*. [53] conducted a study treating 30 patients with GTPS and underlying gluteal tendinosis with either ultrasound-guided percutaneous tendon fenestration (*N* = 15) or autologous PRP injection (*N* = 15). They concluded that tendon fenestration and PRP injections were not statistically different, but both treatment methods showed short-term improvements in pain and function. With low patient recruitment, this study may not have been adequately powered to deduce the results. Despite clinical improvements following treatment with PRP, further research should be conducted to determine the long-term, clinical effectiveness of PRP compared to standard of care. Additionally, further studies should aim to increase patient recruitment for adequate power to determine the effects of PRP in treating GTPS and tendinopathy of the gluteus medius/minimus, specifically.

### Hamstring tendinopathy and injury

Similar to gluteal pathology, there is a paucity of research examining the intraoperative effects of biological therapeutic methods. However, few studies have discussed return to play (RTP) following nonoperative management with the use of PRP injections. A Hamid *et al*. [54] performed an RCT investigating the use of a single PRP injection in the treatment of grade 2 hamstring muscle injuries and its impact on time to RTP. The study found that patients who received a single autologous PRP injection combined with rehabilitation had a significantly lower time to RTP and lower pain severity scores than those using a rehabilitation program alone. Despite clinically significant improvements and shorter time to play, this study examined only 24 patients split between both groups utilizing rudimentary RTP rates for their outcome measurement. Further outcome assessment with standardized outcome measures and follow-up periods are important to consider the effects of PRP injections in this patient population.

On the other hand, some studies have questioned the benefits of PRP injections. A 2019 pilot study comparing image-guided PRP injections and steroid injections had similar short-term results as the aforementioned study. They found that the patients treated with PRP (*N* = 32) had achieved more favorable self-reported pain reduction at 1-week follow-up compared to the steroid group (*N* = 24); however, at 4-week follow-up, the difference between the two groups was not statistically different [55]. Another 2019 cohort study found that ultrasound-guided PRP injections had no significant change in patient-perceived pain scores in 29 patients and 69% of patients reported no change in RTP activity at 8-week follow-up [56]. It is important to identify the limitations inherent with a pilot study as well as a small cohort study, including adequate power, effect sizes associated with the treatment method and larger cohorts. Future research should identify a larger subset of patients while investigating longer follow-up to identify the positive effects of PRP injections for proximal hamstring tendinopathy. While it is important to further investigate the potential benefits of biological therapies in the long-term, more evidence is necessary to also determine the short-term benefits in hamstring injury research beyond the paucity of data available in the current literature.

## CONCLUSION

Despite lack of clear understanding, the clinical use of biologics has increased in the United States. With the rise in clinical use, the purpose was to review the literature regarding the application and efficacy of biologics for common hip conditions. Our review of the literature examined the benefits of PRP, BMAC and HA in the following hip conditions: hip OA, FAIS and associated labral tear pathology, avascular necrosis of the femoral head and gluteal/hamstring tendinopathy. Within the current orthopedic literature, we suggest that there is support for the implementation of biologics to relieve pain and improve function for hip conditions. However, it is important for continued investigation to determine the extent of outcome improvement attributed solely to each biologic therapeutic treatment within hip preservation.
